# Development of a nanoparticle-assisted PCR assay for detection of bovine respiratory syncytial virus

**DOI:** 10.1186/s12917-019-1858-0

**Published:** 2019-04-11

**Authors:** Zhankui Liu, Jianyou Li, Zeyu Liu, Jiawei Li, Zhijie Li, Chao Wang, Jianke Wang, Li Guo

**Affiliations:** 1 Institute of Special Animal and Plant Sciences, Chinese Academy of Agricultural Sciences, Changchun, 130112 China; 2 Graduate School of Northeast Agricultural University, Harbin, China; 3 Antu Animal Husbandry and Veterinary Station, Yanbian, China; 4 Graduate School of Jilin Agricultural University, Jilin, China

**Keywords:** Nanoparticle-assisted PCR assay, Bovine respiratory syncytial virus, Detection

## Abstract

**Background:**

Bovine respiratory syncytial virus (BRSV) is a common pathogen causing respiratory disease in cattle and a significant contributor to the bovine respiratory disease (BRD) complex. BRSV is widely distributed around the world, causing severe economic losses. This study we established a new molecular detection method of BRSV pathogen NanoPCR attributed to the combination of nano-particles in traditional PCR (Polymerase chain reaction) technology.

**Results:**

In this study, the BRSV NanoPCR assay was developed, and its specificity and sensitivity were investigated. The results showed that no cross-reactivity was observed for the NanoPCR assay for related viruses, including the infectious bovine rhinotracheitis virus (IBRV), bovine viral diarrhea virus (BVDV), and bovine parainfluenza virus type 3 (BPIV3), and the assay was more sensitive than the conventional PCR assay, with a detection limit of 1.43 × 10^2^ copies recombinant plasmids per reaction, compared with 1.43 × 10^3^ copies for conventional PCR analysis. Moreover, thirty-nine clinical bovine samples collected from two provinces in North-Eastern China, 46.15% were determined BRSV positive by our NanoPCR assay, compared with 23.07% for conventional PCR.

**Conclusions:**

This is the first report to demonstrate the application of a NanoPCR assay for the detection of BRSV. The sensitive and specific NanoPCR assay developed in this study can be applied widely in clinical diagnosis and field surveillance of BRSV infection.

## Background

Bovine respiratory syncytial virus (BRSV) [1] is a significant cause of respiratory disease and a major contributor to the bovine respiratory disease (BRD) complex [2]. Nanoparticle-assisted polymerase chain reaction (NanoPCR) is a recently developed technique for the rapid detection of bacterial and viral nucleic acid. In the 2–6 month-old calves, the incidence of BRSV infection is high, and the mortality rate is low, secondary bacterial infection, the mortality rate can reach more than 20% [3], the symptoms of re-infection is not apparent after healing the infected calves, the virus can also isolate on cattle without clinical symptoms. Temperature drop or long-distance transport and other stress conditions can also cause outbreaks [4].

In most cases, BRSV can exist for a long time in a herd [5]. However, the specific mechanism is not precise and needs to be studied systematically. The current diagnostic methods for BRSV are primarily enzyme-linked immunosorbent assay (ELISA) and real-time PCR (RT-PCR) [6–8]. Due to the low and unstable replication of the virus, the sensitivity of the previously-mentioned methods is insufficient and often leads to false negative results. Thus, proper diagnosis of the disease is imperative to prevent misdiagnose of the disease and treatment can be given to affected herd. There is still a lack of systematic research on BRSV even though there is a rapid development of the cattle industry in China. Therefore, it is urgent to establish a molecular detection method for BRSV that is efficient, rapid, specific and reproducible.

In 2005, Li et al. [9] reported that nanoparticles (Au nanoparticles, AuNPs) can be used as novel PCR additives to inhibit the production of nonspecific bands in the reamplification system, which can significantly improve nonspecific amplification, wide temperature range is useful, suggesting that nano-gold can produce similar single-stranded binding protein (single-stranded DNA binding protein, SSB) effect and improve the specificity of PCR. Then, Li et al. found that nano-gold can enhance the sensitivity and reaction rate of PCR. The excellent thermal conductivity of nano-gold is the basis for the optimization of nanoparticles. They believe that the role of nano-gold to improve the rapid rise/fall PCR system thermal conductivity, which facilitates the template and primer to be more efficiently matched, NanoPCR technology has also become a new type of PCR technology [10]. Compared with traditional PCR, NanoPCR can improve the sensitivity of PCR detection. Also, NanoPCR saves time more than conventional PCR.

Due to the low sensitivity and low efficiency of the existing molecular detection methods of bovine respiratory syncytial virus (BRSV), We hoping to establish a high sensitivity, simple, rapid and efficient method for detection of BRSV by using nano-gold and PCR molecular detection technology to realize the rapid diagnosis of BRSV, which is of great significance to disease control.

## Results

### Optimization of BRSV NanoPCR assay conditions

**Fig. 1:**
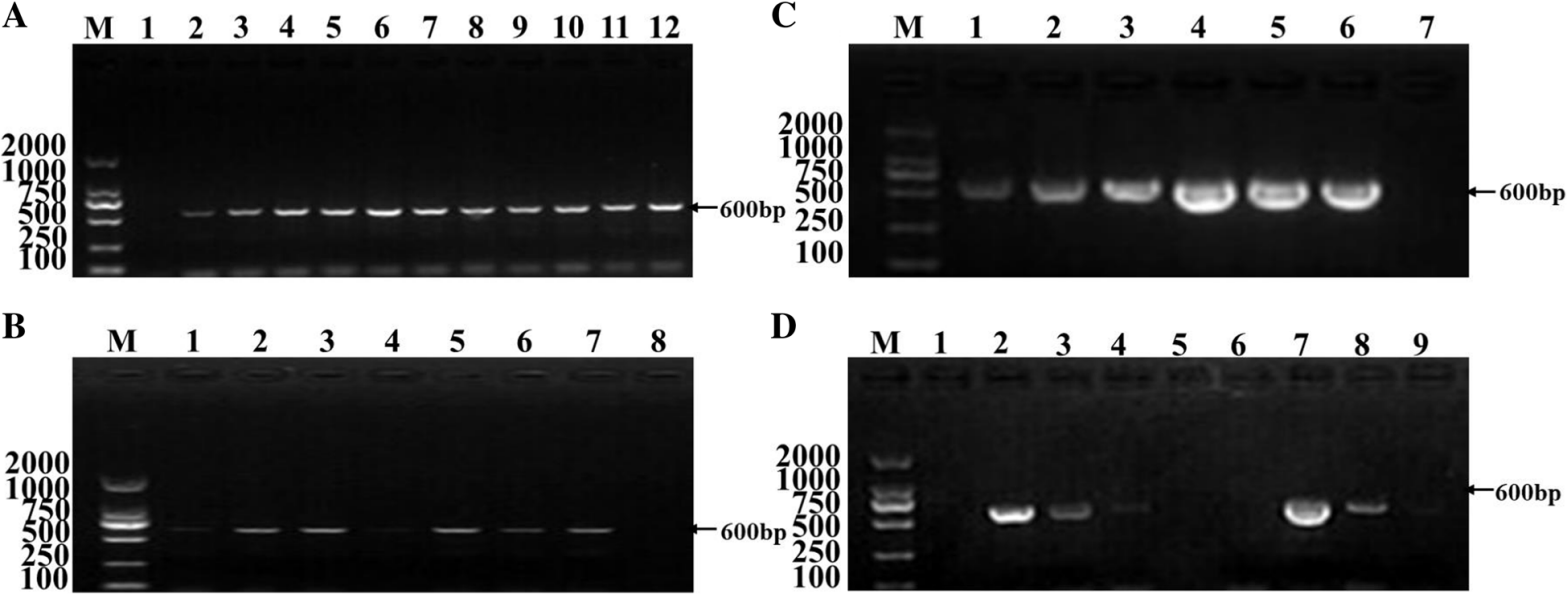
Optimization of annealing temperature (**a**), the volume of 23 nm gold nanoparticles (**b**), the diameter of gold nanoparticles (**c**), and an annealing time of NanoPCR compare with RT-PCR for BRSV NanoPCR. Lane M: DL2000 DNA Marker (Takara); (**a**) Lane 1: Negative control, Lanes 2–12: The annealing temperatures were 51 °C to 61 °C in increments of 1 °C. (**b**) Lanes 1–7: The gold nanoparticles volumes were 0.3 μL to 1.5 μL in increments of 0.2 μL, Lane 8: Negative control. (**c**) Lanes 1–3 and 4–6: The diameters of gold nanoparticles were 20 nm, 23 nm, and 40 nm for RT-PCR and NanoPCR, respectively. Lane 7: Negative control. (**d**) Lanes 2–4 and 7–9: The annealing times were 15 s, 10 s, and 5 s for NanoPCR and RT-PCR, respectively. Lane 1 and 6: Negative control, Lane 5: Blank hole

The optimization of NanoPCR reaction was performed by using BRSV 391–2 strain. Optimized conditions included the optimal concentration of primers, optimal concentration, the diameter of gold nanoparticles, and optimal annealing temperature and time. Using optimized parameters, the NanoRCR amplified BRSV fragment was 600 bp in size. The sequence analysis also showed high similarity (100%) between the products obtained with the NanoPCR amplification of the N gene for BRSV (the objective sequences) and the reference sequence of BRSV. It was found that the band density was optimal at the 55 °C annealing temperature, which was selected for subsequent studies (Fig. 1a). Using this annealing temperature, it was found that the band density was the highest when the gold nanoparticle volume and diameter of nanoparticle were 0.7 μL (Fig. 1b), and 20 nm, 23 nm and 40 nm (Fig. 1c) respectively. In addition, when the annealing time of NanoPCR was reduced to 5 s, the experiment is still valid (Fig. 1d), but the RT-PCR is not valid. This result provides the basis for the rapid diagnosis.

Based on the optimization results, an optimal 25 μL reaction volume was established, containing 2.5 μL 10 × buffer, 2 μL dNTP, 1.5 μL Mg^2+^, 0.5 μL Taq DNA polymerase (TAKARA), 0.7 μL gold nanoparticles, 1.0 μL of extracted DNA or standard plasmid, 0.5 μL of each of forward and reverse primer (10 μM) and ddH_2_O up to 25 μL. The reaction conditions were as follows: 94 °C for 5 min and 35 cycles of 94 °C for 30 s, 57 °C for 5 s, and 72 °C for 30 s, with a final extension for 5 min at 72 °C.

### Specificity of the BRSV NanoPCR assay

**Fig. 2:**
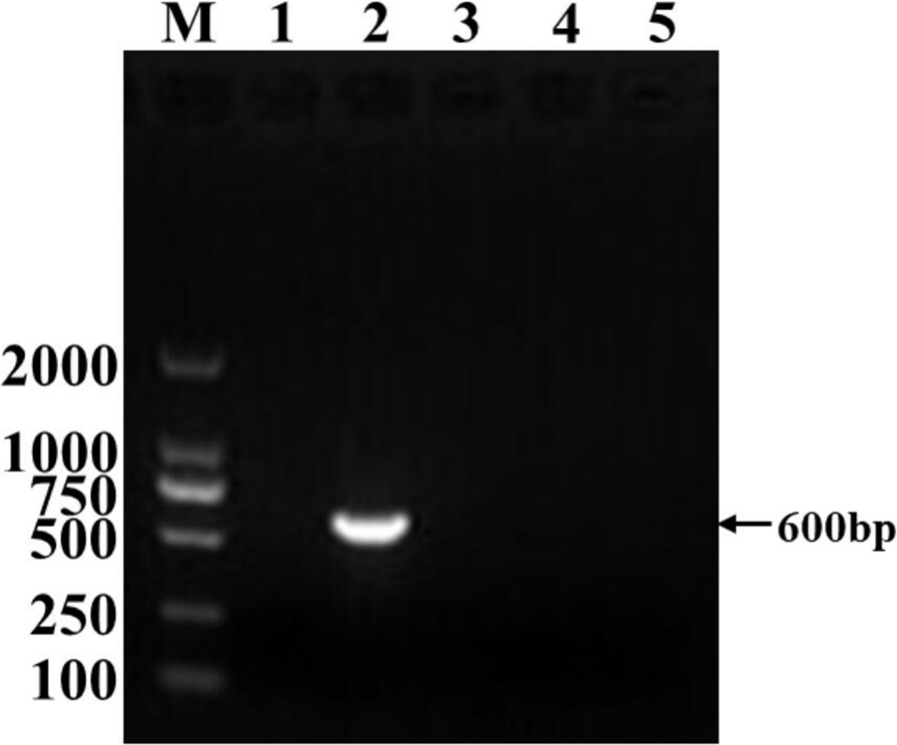
Specificity of the BRSV NanoPCR assay. Lane M: DL2000 DNA Marker (Takara); Lane 1: Negative control; Lane 2–5: The standard positive plasmids of BRSV, IBRV, BIPV3, and BVDV, respectively

Agarose gel electrophoresis analysis showed that no cross-reactivity was observed for related viruses by NanoPCR and conventional PCR, including infectious bovine rhinotracheitis virus (IBRV), bovine viral diarrhea virus (BVDV), and bovine parainfluenza Virus Type 3 (BPIV3) (Fig. 2). The result indicated that the NanoPCR method for the detection of BRSV is specific.

### Sensitivity of the BRSV NanoPCR assay

**Fig. 3:**
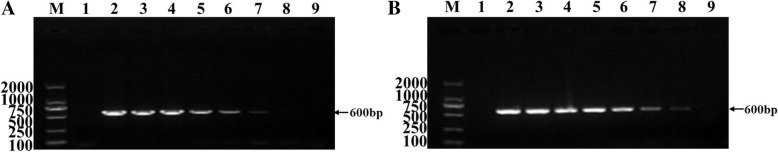
Sensitivity of RT-PCR (**a**) and NanoPCR (**b**) for the detection of BRSV plasmid DNA. Lane M: DL2000 DNA Marker (Takara); Lane 1: Negative control, Lane 2–9: different BRSV plasmid DNA copies subjected to NanoPCR and RT-PCR (1.43 × 10^8^, 1.43 × 10^7^, 1.43 × 10^6^, 1.43 × 10^5^, 1.43 × 10^4^, 1.43 × 10^3^, 1.43 × 10^2^, and 1.43 × 10^1^ copies/μL, respectively)

The sensitivity of BRSV NanoPCR reaction was determined. The detection limit of BRSV NanoPCR assay (1.43 × 10^2^ copies/μL, Fig. 3b) was 10-fold more sensitive than conventional assay (1.43 × 10^3^ copies/μL, Fig. 3a).

### Detection of BRSV in clinical samples

**Fig. 4:**
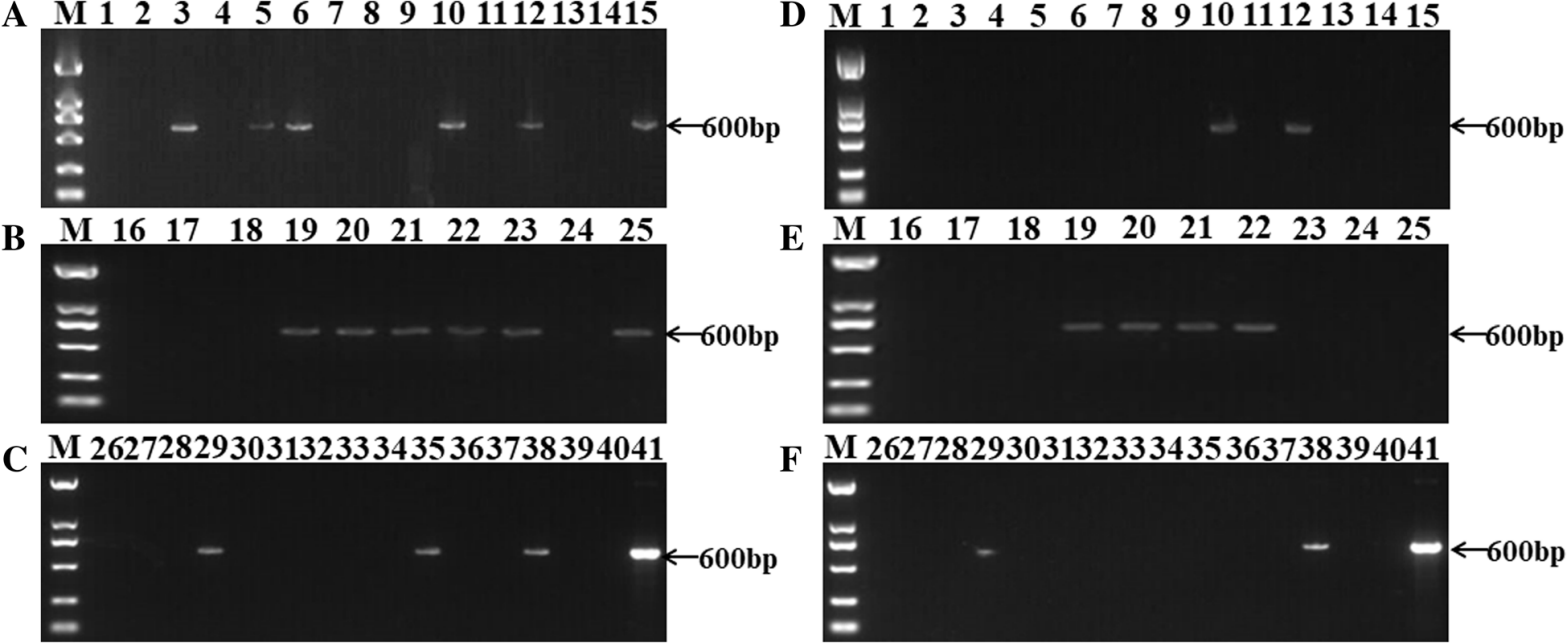
Detection of BRSV in clinical samples by NanoPCR assay (**a**, **b**, **c**) and RT-PCR (**d**, **e**, **f**). Lane M: DL2000 DNA Maker (Takara, Dalian, China); Lane 1 negative control; Lane 41: BRSV plasmid DNA as a template; lanes 2–25: cDNA from clinical nasal swabs samples as the template; lanes 26–40: cDNA from clinical fecal samples as the template

BRSV NanoPCR and conventional PCR were used in clinical samples. Eight (20.5%) of the thirty-nine samples tested by NanoPCR and conventional PCR were positive, and twenty-four (61.5%) were negative by NanoPCR and conventional PCR. Seven samples (17.9%) positive for NanoPCR were negative by conventional PCR, whereas no samples negative for NanoPCR were found to be positive by conventional PCR. Twelve of the fifteen samples tested positive for NanoPCR came from nasal swabs, while six of the eight positive results detected by conventional PCR came from nasal swabs. All the other positive samples came from feces. The NanoPCR random products of five positive sample were subjected subsequently to automated sequencing reactions. The sequence analysis showed high similarity (100%) between the products obtained with the NanoPCR amplification and the reference sequence of BRSV. Clinical samples detection by BRSV NanoPCR were shown in Fig. 4.

## Discussion

In 1970, Paccaud et al [11] first identified BRSV in Europe. In 1974, it was again identified in the United States [12, 13]. It is still an important factor in BRD. The virus is transmitted through nasal secretions and may survive due to the heterogeneity of its genome and the low fidelity of replication. IHC (Immunohistochemistry) and RT-PCR are used to identify viral antigens and viral genome in field specimens easily [14]. The virus is unstable and it is often impossible to try to separate it from clinical samples in the laboratory. Due to rapid turnaround time, RT-PCR has become increasingly popular as a means of identifying BRSV in clinical cases [14]. In 2005, Li et al. [9] showed an excellent optimization effect by adding gold nanoparticles with a diameter of 10 nm and a concentration of 0.4 nm to the PCR system. It is pointed out that the concentration of colloidal gold plays a crucial role in the specificity of PCR. When the concentration of colloidal gold is within the appropriate range (0.2–0.8 nm), the number of nonspecific bands is less and less. The target band is gradually enhanced, and a single bright target band can be obtained. However, when the gold nanoparticles were in excess, the phenomenon of inhibiting the amplification was shown [15].

There are many methods to detect BRSV, including RT-PCR, fluorescence quantitative PCR, ELISA and immunofluorescence antibody detection [16–19]. However, due to antibody reactions, these serological techniques do not distinguish between vaccines and natural infections of wild-type viruses. Besides, although conventional PCR has been used to identify BRSV infection [7], it is time-consuming and insensitive and is not suitable for detecting low viral load in clinical samples. Besides, although LAMP (Loop-mediated isothermal amplification) detects simple [8], it is very vulnerable to contamination. Fluorescent quantitative PCR is more accurate but requires sophisticated equipment and complicated procedures. There is a high false positive rate of ELISA detection methods, and the test of the immunofluorescence antibody takes a long time, and the operation is cumbersome. In recent years, in the field of molecular biology, NanoPCR has become a new type of PCR technique that is more sensitive than traditional PCR. It has been found that NanoPCR thermal cycling can achieve target temperatures faster because of the increased thermal conductivity of gold nanoparticles suspended in the reaction liquid. This faster process of reaching the target temperature helps to enhance reaction specificity and increase product amplification [15, 20]. The use of nanostructured PCR reaction is a new research direction.

This study shows that our NanoPCR detection is an effective and time-saving BRSV detection method. It saves up to 7–10 min compared to conventional PCR. The sensitivity of the method is ten times higher than that of conventional PCR and this assay is specific to BRSV. Moreover, there is no cross-reactivity to other viruses. In summary, we have developed a convenient NanoPCR method for rapid, sensitive and specific detection of BRSV.

## Conclusion

This study we established an efficient, fast, specific, reproducible detection method of BRSV pathogen NanoPCR attributed to the combination of nano-particles in traditional PCR technology. The established BRSV Nano PCR assay can effectively solve the problem of the low titer of BRSV, low detection sensitivity and rate of clinical samples, it also has good specificity and ten times higher sensitivity than conventional PCR, clinical testing efficiency is equally much higher than RT-PCR. Preliminary application based on clinical sample testing. The developed method can be applied to the field of molecular detection of BRSV. It will play an active role in the detection of epidemic diseases, epidemic prevention, purification, and biosafety, to realize the rapid diagnosis of epidemic diseases. It also has essential meanings for both early detections of subclinical infection and disease control.

## Methods

### Viruses and clinical samples

The standard positive plasmids (BRSV, BVDV, IBRV, BPIV3) in this study have been described in the previous study [21]. The thirty-nine clinical samples were obtained from different cattle farms in Jilin and Heilongjiang provinces. All samples from our laboratory were saved previously. The samples were taken from the feces and nasal swabs outside the body of the experimental animals, and the whole experiment did not cause harm to the animals.

### Viral DNA/RNA extraction

Extraction of viral RNA from 200 μL of pure BRSV culture or tissue samples using E.Z.N.A.™ Viral RNA Kit (OMEGA Inc., Dusseldorf, Germany) following the manufacturer’ instruction. BRSV, BVDV and BPIV3 RNA reverse transcription using reverse transcription kit (TransGen Biotech, AT311–03) following the manufacturer’ instruction. The extracts were aliquoted and stored at − 80 °C.

### PCR primer

The common BRSV N gene sequence (GenBank: S40504.1) was obtained by comparing the genomes of different BRSV isolates collected from publicly available sequence data. Primers were selected and designed from conserved N genes using PRIMER PREMIER 5.0 software (Molecular Biology Insights, Inc., Cascade, CO, USA) to produce 600 bp amplicon (Table 1).

**Table 1:** NanoPCR and RT-PCR primers used for amplification of BRSV

Primer name	Length (nt)	Sequence (5′-3′)	Tm (°C)	Product (bp)
P1	19	TATGCTATGTCCCGATTGG	55.4	600
P2	21	ACTGATTTGGCTAGTACACCC	58.0	

### Conventional PCR

Routine PCR analysis of BRSV was performed using primer group to produce the PCR product with a predicted length of 600 bp. PCR was carried out in 25 μL reaction volume of 1 μL extracted DNA or 0.5 μL standard plasmid, 2.5 μL 10 × buffer, 2 μL dNTP, 1.5 μL Mg^2+^, 0.5 μL Taq DNA polymerase (TAKARA), and 0.5 μL of each of forward and reverse primer (10 μM) by following the manufacturer’s protocol with the following cycling times and temperatures: 94 °C for 5 min and 35 C cycles of 94 °C for 30 s, 57 °C for 15 s, and 72 °C for 30 s, with a final extension for 5 min at 72 °C. PCR was carried out in a Life Express Thermal Cycler (HANGZHOU BIOER TECHNOLOGY CO., LTD, China) and the products were analyzed by 1% agarose gel [22].

### Optimization of BRSV NanoPCR assay conditions

BSRV NanoPCR optimized the annealing temperature, time, concentration and diameter of gold nanoparticles (SIGMA) with the same primer pairs as conventional PCR. The annealing temperature range of Life Express thermal cycler instrument ranges from 51 °C to 61 °C. The diameter of gold nanoparticles is 20 nm, 23 nm, and 40 nm, the volume range from 0.5 to 1.5 μL in increments of 0.2 μL by 20. Products were visualized on 1% agarose gels at a voltage of 180 V for 20 min. ImageJ 1.46r software (National Institutes of Health, Bethesda, MA, USA) was used for quantitative gel analysis of all bands.

### Specificity of BRSV NanoPCR assay

The NanoPCR reactions with different viral nucleic acids (including BRSV, BVDV, BPIV3, and IBRV) were performed using optimized reaction parameters to determine the specificity of the NanoPCR assay.

### Sensitivity of BRSV NanoPCR assay

The detection limit of the BRSV NanoPCR assay was compared to the detection limit of conventional PCR using a 10-fold dilution series of BRSV standard positive plasmids (ranging from 1.43 × 10^8^ to 1.43 × 10^1^ copies/μL), and ddH_2_O was used as a negative control. The PCR product was electrophoresed on a 1% agarose gel.

### Detection of BRSV in clinical samples

The thirty-nine clinical samples included feces, and nasal swabs were collected from different cattle farms in Jilin and Heilongjiang provinces. All samples came from our laboratory saved previously. The NanoPCR and conventional PCR assays were performed simultaneously on the thirty-nine clinical samples. Part of the positive samples was sent to sequence by Comate Biosciences Co., Ltd.

## Abbreviations

AuNPs: Au nanoparticles
BPIV3: Bovine parainfluenza virus type 3
BRD: Bovine respiratory disease
BRSV: Bovine respiratory syncytial virus
BVDV: Bovine viral diarrhea virus
CAAS: Chinese Academy of Agricultural Sciences
ELISA: Enzyme-linked immunosorbent assay
IBRV: Infectious bovine rhinotracheitis virus
IHC: Immunohistochemistry
LAMP: Loop-mediated isothermal amplification
PCR: Polymerase chain reaction
RT-PCR: Real-time PCR
SSB: Single-stranded DNA binding protein
